# Developing a Traditional Chinese Medicine-Based Lifestyle Content Framework for Insomnia: A Modified Delphi Study

**DOI:** 10.3390/healthcare14182907

**Published:** 2026-09-08

**Authors:** Shirong Wu, Man Ling Ng, Yan Yi Fong, Fiona Yan Yee Ho, Jia Yin Ruan, Na Zhang, Ka Ying Heidi Lo, Hai Yong Chen, Dennis Chak Fai Ma, Danny Jucheng Yu, Wing Fai Yeung

**Affiliations:** 1 School of Nursing, The Hong Kong Polytechnic University, Hong Kong SAR, China; 25073009r@connect.polyu.hk (S.W.);; 2 Department of Psychology, The Chinese University of Hong Kong, Hong Kong SAR, China; 3 Rory Meyers College of Nursing, New York University, New York, NY 10010, USA; 4 Department of Psychiatry, The University of Hong Kong, Hong Kong SAR, China; 5 School of Chinese Medicine, The University of Hong Kong, Hong Kong SAR, China; 6 School of Chinese Medicine, The Hong Kong Baptist University, Hong Kong SAR, China

**Keywords:** consensus, expert, TCM, sleep, survey, modified Delphi technique

## Abstract

**Highlights:**

**What are the main findings?**

- Through a four-round modified Delphi process with local panel experts, this study developed a consensus-based Traditional Chinese Medicine-based lifestyle (TCM-L) content framework for insomnia.
- The finalized TCM-L framework not only established the core domains and subordinate items but also determined an implementation priority sequence: (1) introduction to TCM-L, (2) sleep–wake routines, (3) dietary regulation, (4) emotional regulation, and tied at (5) mind–body exercise and acupoint massage.

**What are the implications of the main findings?**

- The framework offers an expert-endorsed, structured foundation to guide subsequent curriculum design, intervention manual development, and standardized health education for insomnia.
- By integrating multifaceted lifestyle components into everyday routines, the framework empowers individuals with insomnia to adopt accessible, self-directed daily health preservation practices for long-term sleep self-management.

**Abstract:**

**Background:** Insomnia requires long-term self-management, prompting many patients to turn to Traditional Chinese Medicine-based lifestyle (TCM-L) practices for health preservation. However, a consensus-based TCM-L content framework is lacking. This study aims to develop a consensus-based TCM-L content framework using a modified Delphi method. **Methods:** A modified Delphi study was conducted in two phases. In phase 1, a preliminary content framework was developed based on a systematic review, TCM textbooks, and clinical guidelines, consisting of 10 initial domains and 82 subordinate items. In phase 2, 18 local Chinese medicine practitioners were purposively recruited to evaluate the framework over four iterative rounds. Items were rated on a 5-point Likert scale, with consensus defined as a mean ≥ 4.0, a coefficient of variation < 0.25, and a consensus level of agreement ≥ 80%. Iterative modifications, deletions, and additions were informed by both these numerical thresholds and qualitative expert feedback. Kendall’s *W* was calculated to assess the coordination of consensus. **Results:** All 18 experts completed four rounds (100% response rate) with high authority (*Cr* = 0.864–0.883). The initial 10 domains and 82 subordinate items were refined to six domains and 49 subordinate items. Expert consensus was statistically significant across rounds (*p* < 0.01), with Kendall’s *W* demonstrating a high concordance of 0.717 for domain importance in the final round. The prioritized sequence of the six domains in the final TCM-L framework was: (1) Domain 1: introduction to TCM-L, (2) Domain 4: sleep–wake routines, (3) Domain 3: dietary regulation, (4) Domain 2: emotional regulation, and tied at (5) Domain 5: mind–body exercise and Domain 6: acupoint massage. **Conclusions:** This study established a consensus-based TCM-L content framework for insomnia, providing a culturally relevant foundational structure for future lifestyle interventions, clinical education, and self-management support.

## 1. Background

Insomnia is characterized by difficulty initiating or maintaining sleep, early-morning awakening, and daytime impairment [1]. Worldwide, approximately 10–16% of adults meet the diagnostic criteria for insomnia disorder [1,2,3], whereas individual insomnia symptoms are substantially more prevalent, affecting roughly one-third of the general population depending on the definition applied [1,4]. Specifically, chronic insomnia is associated with impaired daytime functioning, reduced quality of life, emotional distress, and increased risk of mental and cardiometabolic comorbidity [5,6,7]. Both behavioral and pharmacological treatments are available, but cognitive behavioral therapy for insomnia (CBT-I) is recommended as the first-line treatment for chronic insomnia because it has fewer harms and more durable benefits than pharmacotherapy [8], although implementation in routine care remains limited by cost, limited provider availability, and treatment burden [9,10].

As a result, many patients continue to seek more accessible and acceptable non-pharmacological options. This trend is especially critical in Hong Kong, where at least one insomnia symptom meeting quantitative criteria (defined as difficulty falling asleep, staying awake during the night, or early morning awakening for ≥30 min, occurring ≥ 3 nights per week) affects 40.1% of the adult population (with DSM-5 insomnia disorder affecting 10.8%, as reported by Chung et al.) [11], representing a substantial public health challenge. In addition to practical barriers, psychological interventions may be less acceptable to some patients in the Hong Kong Chinese context because mental health-related stigma, face concerns, and perceived barriers to help-seeking can discourage engagement with psychologically framed treatments [12,13]. In contrast, traditional Chinese medicine (TCM) is often welcomed as a culturally familiar option [14,15], as its health concepts are deeply rooted in local beliefs. Beyond herbal medicine, non-pharmacological TCM modalities, such as Tai Chi, music therapy, and acupoint massage, have been investigated in various clinical studies [16,17,18,19]. However, clinical research often evaluates single modalities in isolation, which may be insufficient for a multifaceted condition driven by interconnected daily behaviors.

According to TCM theory, insomnia stems from systemic disharmonies, specifically: (1) Yin-Yang imbalance, where the failure of Yin to anchor Yang leads to hyperactive mental states at night; (2) Qi-Blood deficiency, which results in inadequate nourishment of the Heart-Spirit; and (3) Zang-Fu organ dysfunction, particularly involving the Heart, Liver, and Spleen, which disrupts the body’s physiological homeostasis [15]. From this holistic perspective, sleep is viewed as a reflection of overall lifestyle. The TCM-based lifestyle (TCM-L) framework focuses on non-pharmacological self-care practices and consists of at least two of the following components: sleep–wake regulation, dietary regulation, emotional adjustment, mind–body exercise, and acupoint self-care. Distinct from general TCM clinical treatment, which relies on syndrome differentiation and professional supervision (such as personalized herbal prescriptions), TCM-L is designed as a structured, adjunctive self-management approach that individuals can safely integrate into their daily lives [12]. Therefore, establishing a consensus-based lifestyle content framework is essential to provide culturally tailored, accessible options for individuals with insomnia symptoms [20].

An extensive review of the literature on TCM-L interventions for insomnia revealed that although current studies have demonstrated their clinical potential, these interventions are often based on inconsistent clinical experiences or diverse classical texts and lack a standardized structure. Within the scope of our review, no comparable consensus-based framework was identified that defines the core domains or prioritizes or sequences these components. This lack of standardization limits the reproducibility of research and hinders the systematic implementation of structured self-care. Establishing a consensus-based TCM-L content framework is therefore essential to bridge this gap. A modified Delphi approach is well-suited for this purpose, as it enables the systematic synthesis of local expert opinions to build consensus on complex health content [21]. Accordingly, this study aims to develop a locally grounded, expert-consensus content framework for individuals with insomnia symptoms, providing a foundational structure to guide the development and evaluation of subsequent interventions.

## 2. Methods

### 2.1. Study Design

**Figure 1:**
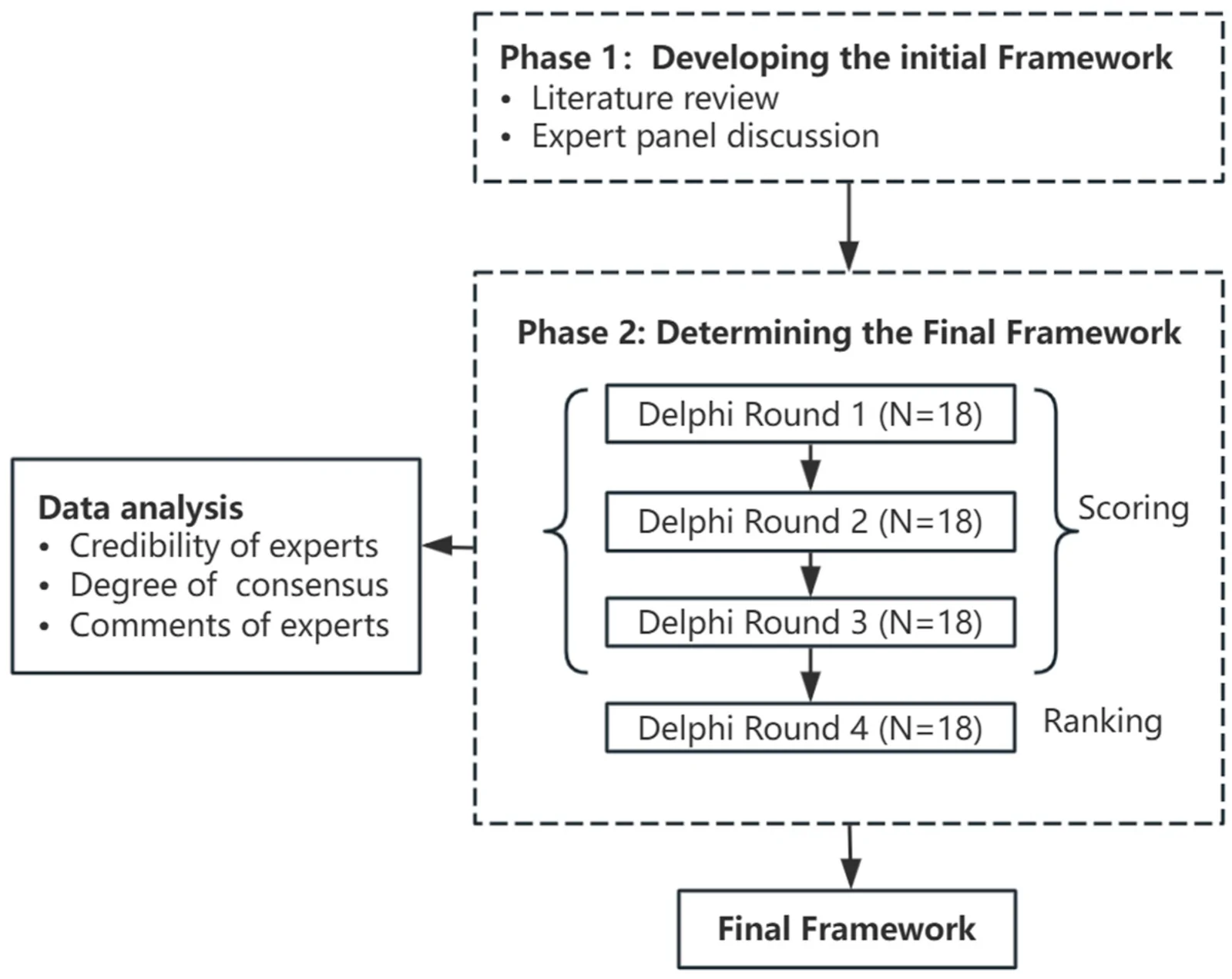
Research design flowchart.

As illustrated in Figure 1, this study employed a modified Delphi method to develop a consensus-based content framework [21], reported in accordance with the Guidance on Conducting and Reporting Delphi Studies (CREDES) [22]. Unlike a conventional Delphi process—which typically utilizes an open-ended first round for item generation—this study utilized a preconstructed item pool to streamline the consultation. The study was conducted in two phases. Phase 1 involved a comprehensive review of relevant literature (including a systematic review, TCM textbooks, and clinical guidelines) and consultations with a multidisciplinary steering panel to generate and refine the preliminary item pool. Phase 2 consisted of iterative Delphi rounds to achieve a predefined consensus among local Chinese medicine practitioners (CMPs). Specifically, the first three rounds focused on item scoring and consensus evaluation using 5-point Likert scales, whereas the final round involved expert ranking to establish the relative importance of items and the implementation sequence of the core domains. Ethical approval was obtained from the Human Subjects Ethics Sub-committee of the Hong Kong Polytechnic University (Ref: HSEARS20251028001) prior to the study’s commencement. Informed consent was obtained from all experts before participation.

### 2.2. Phase 1: Development of Initial TCM-L Content Framework

#### 2.2.1. Organization of Working Group

The research team consisted of five members. The principal investigator, who is a registered CMP (W.F.Y.), oversaw the overall planning of the study. A postdoctoral researcher provided guidance on the research process (J.Y.R.), and the three other research members (S.W., M.L.N., and Y.Y.F.) were responsible for the study management and implementation.

#### 2.2.2. Generation of Preliminary Content Framework

The preliminary TCM-L Content Framework was synthesized from three primary sources: (1) a systematic review and meta-analysis previously conducted by the research team (PROSPERO: CRD420251140366), which included 14 articles from which specific lifestyle intervention components and characteristics were extracted via qualitative content extraction [23,24,25,26,27,28,29,30,31,32,33,34,35,36] (the flowchart of study selection is provided in Supplementary Figure S1); (2) 2 authoritative TCM textbooks related to insomnia [37,38]; and (3) TCM clinical practice guidelines for insomnia [15,39,40].

#### 2.2.3. Expert Panel Discussion

A multidisciplinary steering panel comprising five experts (*n* = 5) was established using purposive sampling to ensure the accuracy and clinical relevance of the preliminary TCM-L Content Framework. The panel consisted of a senior Chinese medicine practitioner, a general nurse, a mental health nurse, a psychiatrist, and a clinical psychologist, ensuring a holistic and multidisciplinary perspective. In Phase 1, TCM-L framework components identified from the literature, textbooks, and guidelines were comprehensively extracted and compiled into a preliminary item pool, which was then structured into initial domains. These preliminary items and domain structures were reviewed by the steering panel for clarity, accuracy, and comprehensibility. Any discrepancies, overlapping items, or feedback during this synthesis and review process were resolved through research team discussions and panel consultations until consensus was reached, thereby finalizing the version used for the first round of the Delphi study.

### 2.3. Phase 2: Delphi Rounds to Determine the Final TCM-L Content Framework

#### 2.3.1. Panel of Experts

Registered CMPs with clinical practice were recruited through purposive sampling between November and December 2025 to ensure professional accuracy in the TCM-L content. The inclusion criteria were as follows: (1) experience delivering TCM health preservation for insomnia, (2) at least 5 years of clinical experience as a registered CMP, and (3) commitment to completing multiple survey rounds. Although there is no consensus on the optimal sample size for a Delphi study, the literature suggests that a panel of 15 to 30 experts is sufficient to ensure consensus stability and diversity of perspectives [41]. This study initially invited 18 experts after considering that candidates were selected from various settings, including academic institutions, non-governmental organizations, private practices, and government-subsidized hospital authority TCM clinics in Hong Kong, to ensure broad representation.

#### 2.3.2. Data Collection

The Delphi consultation was conducted between January and April 2026. For the first round, the survey questionnaire consisted of three parts. (a) Introduction: This section briefly explained the study’s purpose, voluntary nature, and confidentiality procedure. (b) Preliminary TCM-L content framework: This section included 82 items and 10 domains proposed in phase 1. Experts were asked to rate the importance of each item on a 5-point Likert scale (from 1 = not at all important to 5 = very important) and encouraged to provide qualitative comments or suggestions. (c) Demographic characteristics: This section included basic information about the experts, including age, gender, professional experience, and work setting. The familiarity coefficient (*Cs*) and judgment coefficient (*Ca*) were collected to assess expert authority [42]. The *Cs* data were separated into five levels (0.20 = unfamiliar, 0.4 = less familiar, 0.6 = generally familiar, 0.8 = very familiar, and 1.0 = extremely familiar), and the *Ca* data were divided into four categories: theoretical analysis, literature reference, practical experience, and subjective judgment.

Delphi questionnaires were distributed and collected individually via email, with experts requested to complete each round within 10 days. To ensure independent judgment and minimize social pressure, strict quasi-anonymity was maintained throughout the process: panelists remained entirely anonymous to one another while their identities and individual responses were known to the research team for administrative coordination. In each round, experts rated the items and received a summary of feedback from the preceding round. Items were iteratively revised, deleted, or added based on predefined consensus criteria and qualitative expert feedback. This iterative scoring process continued until a stable consensus was achieved across the consensus-rating rounds, operationally defined as all retained items meeting the predefined statistical thresholds without requiring further substantive modifications. Following the consensus-rating stage, a final ranking round was conducted. In this round, experts performed a ranking task to determine item priorities using a lower-is-better scoring system (e.g., a rank of 1 was assigned to the most important item, so the item with the lowest total rank sum had the highest priority).

#### 2.3.3. Data Analysis

Statistical analysis was performed using IBM SPSS Statistics (version 28.0) and MS Excel 2019. Expert positivity was assessed via questionnaire response rates, and the expert authority coefficient (*Cr*) was calculated as the arithmetic mean of *Ca* and *Cs* (*Cr* ≥ 0.70 considered acceptable) [43]. For consensus-building, descriptive statistics included means (*M*), standard deviations (*SD*), and coefficients of variation (*CVs*). The predefined consensus thresholds for item retention were as follows: *M* ≥ 4.0, *CV* < 0.25, and a consensus level of agreement (*CLA*, defined as the proportion of experts who rated an item 4 or 5) ≥80% [44]. Opinion coordination and final ranking consensus were evaluated using the *CV* and Kendall’s coefficient of concordance (*W*). A *p*-value < 0.05 indicated statistical significance. Kendall’s *W* ranged from 0 to 1, with *W* < 0.3 considered weak, 0.3–0.5 moderate, 0.5–0.7 good, 0.7–0.9 strong, and 0.9 unusually strong agreement [45]. Qualitative comments were independently reviewed by two investigators, thematically coded (e.g., wording refinement and item addition/deletion), and discussed until consensus was reached. In the final round, the mean ranks were calculated to determine the relative importance of items within each domain and the overall implementation sequence of the core domains [46]. In cases where two or more items or domains received identical mean ranks or rank sums, they were assigned equal priority (tied ranks) to reflect the experts’ shared consensus on their equivalent importance.

## 3. Results

### 3.1. Expert Demographics

All 18 invited experts (10 females, 55.6%) were registered CMPs in Hong Kong and completed the four rounds of the Delphi consultation, yielding a 100% response rate. Most experts (94.4%) held postgraduate qualifications (master’s or PhD). In terms of clinical experience, the panel showed an even distribution, with six experts (33.3%) each having <10, 10–19, and ≥20 years of practice. Detailed demographics are summarized in Table 1.

### 3.2. Expert Authority and Coordination

The *Cr* across the four rounds ranged from 0.864 to 0.883 (Table 2), indicating a high level of expert authority. Expert engagement was high: all experts completed all questionnaires in each round; 13 experts (72.2%) provided qualitative feedback in round 1; and all 18 experts (100%) contributed suggestions in rounds 2 and 3. By round 4, only five experts offered minor refinements, suggesting that expert consensus had been reached.

The degree of coordination was assessed using the *CV* and Kendall’s *W* (Table 3). The *CV* indicators showed a decreasing trend across the first three rounds (Round 1: 0.08–0.72; Round 2: 0.08–0.32; Round 3: 0.07–0.27), indicating greater consensus. During the scoring rounds (Rounds 1–3), the Kendall’s *W* coefficients for both domains (ranging from 0.194 to 0.372) and items (ranging from 0.180 to 0.318) demonstrated statistically significant agreement (*p* < 0.05). Although a minor fluctuation in Kendall’s *W* was observed in Round 3 due to item refinements and the retention of highly qualified items, Round 4 showed considerable improvement, with Kendall’s *W* reaching 0.717 for domain importance (*p* < 0.001), while the coefficients for items within specific domains ranged from 0.249 to 0.503 (all *p* < 0.01), indicating a weak-to-moderate concordance among panelists regarding item prioritization.

### 3.3. Phase 1: Initial Framework Development

The initial framework submitted for validation contained 10 domains and 82 items (Supplementary Materials Table S1).

### 3.4. Phase 2: Iterative Delphi Consultations

**Round 1**

The mean scores for the 10 domains and 82 items ranged from 3.17 to 4.83. Consensus was reached for six domains and 40 items. Four domains (social, sexual, hobbies, and bathing) were removed because they, along with their constituent items, failed to meet the consensus thresholds. Social adaptability was excluded due to difficulties in operationalization and limited insomnia-specific evidence. Sexual regulation did not reach consensus due to concerns about acceptability and applicability across patient groups. Cultivating hobbies was not retained due to heterogeneity and context-specific dependence. Bathing was removed as an independent domain, although foot soaking before bedtime was retained under “sleep–wake routines”.

Additionally, 12 items failed to reach the consensus criteria: nine were deleted due to a lack of suggested improvements, and three (e.g., bedtime regulation, sleeping postures, and sleep taboos and precautions) were revised and merged based on Delphi experts’ suggestions and retained for the subsequent round survey (Supplementary Materials Table S1). Among the items meeting consensus, two were modified (i.e., revising “cease overthinking” to “transform the mindset” because halting overthinking is unrealistic for patients with insomnia, “tea and soup therapy” to “health-preserving herbal teas” due to the inconvenience of soup preparation), and seven were merged into three items. Furthermore, 12 new items were added based on expert feedback. Notably, domain 10 (meridian regulation) was restructured as “supplementary TCM practices” for round 2 because it contained only three items, with three new items (e.g., foot bath, aromatherapy, and five-tone) added.

**Round 2**

The mean scores for the six domains and 51 items ranged from 3.39 to 4.89 (Supplementary Materials Table S2). Five domains and 41 items met the consensus criteria, whereas one domain (“supplementary TCM practices”) and 10 items did not. Based on expert suggestions to focus on TCM meridians, this domain was revised to “acupoint massage”. Among the 10 items that failed to meet the consensus criteria, four were revised in accordance with Delphi experts’ comments, and six were deleted. For the items that met consensus, three were revised, two were merged into one, and 11 new items were added in accordance with the experts’ suggestions.

**Round 3**

The mean scores for the six domains and 55 items ranged from 3.78 to 4.89 (Supplementary Materials Table S3). Consensus was reached for all six domains and 51 items. Of the four items that failed to reach consensus, two were deleted, one was merged into item 3.4, and one (item 5.7) was retained after revision. For item 5.7, “meditation” was removed due to its perceived difficulty for self-application by patients with insomnia. Additionally, three items were merged into one. Prior to Round 4, items 2.2 and 2.3 were integrated with their sub-items, and a non-scored introductory item defining “acupoint massage” was added following working group discussions to provide conceptual clarity. This item was retained in the finalized framework as contextual guidance (Table 4).

**Round 4**

The final framework, comprising six domains and 49 items (Table 5), was ranked by the expert panel to prioritize items (Supplementary Materials Table S4) and to establish the domain implementation sequence (Supplementary Materials Table S5). Retaining the legacy Domain IDs (Domains 1–6), the expert implementation priority sequence for the six modules was as follows:

(1) Rank 1 (Domain 1: Introduction to TCM-L framework): Prioritized foundational TCM concepts, including definition, etiology, and sleep theories of insomnia, over specific pathogenesis-based therapies, establishing a theoretical baseline for patient self-management.
(2) Rank 2 (Domain 4: Sleep–wake routines): Prioritized the theory of sleep–wake routines and the definition of daily living health preservation, followed by methods for judging sleep quality and formulating personal sleep plans, whereas environmental adjustments and foot soaking ranked lowest.
(3) Rank 3 (Domain 3: Dietary regulation): Prioritized foundational TCM dietary theories and overarching principles (e.g., individualized strategies, moderation, and dietary taboos) over recommendations for specific ingredients, herbal teas, or precautions.
(4) Rank 4 (Domain 2: Emotional regulation): Prioritized the definition of emotional regulation and general self-regulation strategies, followed by catharsis methods, whereas behavioral advice (e.g., avoiding electronic devices before bed) ranked lowest.
(5) Rank 5 (Tied) (Domain 6: Acupoint massage): Led jointly by practical in-class training on key acupoints and the definition/functions of acupoint massage, followed by hands-on naive techniques and *Yongquan* (KI-1) massage.
(6) Rank 5 (Tied) (Domain 5: Mind–body exercise): Prioritized the characteristics, functions, and key essentials of mind–body exercise, followed by principles of practice, before moving to specific behavioral practices such as *Baduanjin* or *Tai Chi*.

## 4. Discussion

This modified Delphi study developed a consensus-based TCM-L framework for insomnia through four iterative rounds of expert consultation, refining an initial 10-domain structure into a focused six-domain model comprising 49 prioritized items. The 100% response rate across rounds, coupled with a high authority coefficient (*Cr* > 0.86) and strong concordance on domain-level importance, indicates robust panel consensus on the overall framework, although concordance for specific item-level rankings was only weak to moderate. By resolving the marked heterogeneity of previous protocols that lacked standardized core components [20,47], this framework establishes an expert-endorsed baseline to guide structured sleep health education and inform future clinical intervention development.

Existing clinical guidelines for insomnia mainly fall into two categories: international guidelines, which predominantly provide recommendations on CBT-I, pharmacotherapy, and sleep hygiene education [39,40,48,49], and representative Chinese medicine guidelines—such as the Hong Kong Chinese medicine clinical practice guideline for insomnia—which offer practitioner-oriented guidance on pattern differentiation, herbal prescriptions, acupuncture, and general health preservation [15,50]. While these guidelines provide essential clinical references, their lifestyle-related recommendations are typically presented as generic, supportive advice designed for broad health education. The present Delphi framework addresses this implementation gap by specifying and prioritizing essential TCM-L components, thereby facilitating the translation of traditional wellness concepts into structured self-care content. In this way, it complements existing clinical guidance and is particularly relevant to community-based insomnia management in regions where TCM concepts are culturally familiar and commonly integrated into daily life.

This final TCM-L content framework differs from general lifestyle medicine (LM) for insomnia, which emphasizes evidence-based self-management across diet, exercise, and sleep and stress management [12]. While both approaches emphasize behavioral change and self-management, TCM-L contextualizes these components within TCM health preservation (*Yang Sheng*), offering structured guidance tailored to individual body constitution (*Ti Zhi*) and syndrome patterns (*Bian Zheng*) [50]. This approach is strongly aligned with recent clinical evidence suggesting that specific constitutional differences significantly influence insomnia severity and symptom burden [51,52], thereby providing a robust rationale for integrating individualization into a standardized lifestyle framework. Furthermore, because TCM views insomnia through a holistic lens where sleep disturbances are intrinsically linked to interrelated lifestyle factors, non-pharmacological management necessitates a comprehensive, multi-dimensional approach. Therefore, the present framework should be conceptualized not as a replacement for LM, but as a provisional guidance structure that enriches general LM with an individualized TCM perspective.

The domain refinements during the Delphi process further clarify the framework’s intended scope and implementation orientation. The excluded domains generally shared features such as high contextual variability, limited insomnia-specific evidence, or difficulty with consistent operationalization as rated by the panel under the study’s consensus criteria. This suggests that the final framework does not attempt to represent the full breadth of TCM health preservation, but instead prioritizes components that are deemed by experts to be sufficiently well-defined, acceptable, and compatible with home-based self-management. The refinement of meridian-related practices into self-acupoint massage also reflects this orientation. Many acupuncture- or meridian-based practices require practitioner delivery or specific training, making them less suitable for a structured self-management program [53,54]. However, self-administered acupressure has shown potential benefits and may even be comparable to acupuncture for insomnia [55], supporting the inclusion of acupoint-based self-care within the acupoint massage domain [56]. Overall, these refinements indicate that the framework balances TCM theoretical relevance with practical feasibility, which warrants empirical evaluation in future studies.

The Round 4 domain rankings further evaluated the relative priority of the six modules within the framework. The modules were ordered based on expert consensus as follows: “introduction to TCM-L framework” (Rank 1), followed by “sleep–wake routines,” “dietary regulation,” and “emotional regulation.” Notably, “acupoint massage” and “mind–body exercise” achieved identical rank sums and mean ranks (both with a mean rank of 4.78), reflecting a statistical tie in expert prioritization rather than a definitive sequential separation. This overall progression—moving from foundational understanding to daily lifestyle adjustment and specific self-care techniques—reflects how TCM approaches to insomnia are prioritized by experts, typically emphasizing broader regulation of daily routines, diet, and emotional well-being before technique-based practices [20,47]. The later placement of acupoint massage and mind–body exercise may reflect their greater instructional demands and the need for sustained participant engagement, despite evidence supporting their benefits for insomnia [57]. This sequencing also suggests that different domains may require different modes of delivery. Introductory content and sleep–wake routines may be well suited to didactic education and self-monitoring, whereas acupoint massage and mind–body exercise may require demonstration, guided practice, and home rehearsal. Dietary and emotional regulation may further benefit from individualized discussion, particularly when adapted to constitutional or pattern-based considerations [58].

### 4.1. Strengths and Limitations

This study has several strengths. First, the framework was developed through a structured four-round modified Delphi process utilizing predefined consensus criteria, achieving complete panel retention and enabling iterative refinement from an initial broad item pool to a consensus-based structure. Second, the final framework includes not only core content domains and items but also item importance rankings and domain sequencing, thereby providing structured guidance for content emphasis and curriculum organization.

However, several limitations should be acknowledged. First, the formal Delphi panel relied on purposive sampling and included exclusively registered CMPs in Hong Kong. While this ensured regional contextual relevance, it resulted in a professionally homogeneous panel whose shared TCM-based assumptions may not fully represent patients, sleep specialists, behavioral intervention experts, or practitioners outside the local context. Second, given the focus on establishing the initial framework, patient perspectives were not systematically incorporated during the formal consensus rounds; consequently, claims regarding cultural suitability, comprehensibility, acceptability, and usability require direct empirical assessment among the intended end users. Third, the framework primarily defines the content and structural domains of the intervention. Specific statements regarding safety precautions, contraindications, and constitution self-evaluation represent expert-endorsed framework content rather than empirically validated clinical recommendations, and delivery procedures as well as dosage require further empirical evaluation and specification in subsequent trials. Fourth, the initial item pool partly derived from regional Chinese-language literature of variable methodological rigor; although the iterative Delphi process helped mitigate potential bias, it may not completely eliminate underlying quality constraints.

### 4.2. Future Research

Building upon these findings, subsequent research should follow a structured pathway: (1) co-developing and refining course teaching materials with intended patients and multidisciplinary experts (e.g., sleep specialists and behavioral intervention experts); (2) operationalizing the framework into a standardized intervention manual specifying delivery modes, dosage, and safety protocols; (3) rigorously evaluating comprehensibility, acceptability, cultural relevance, feasibility, adherence, and safety before clinical effectiveness testing; and (4) examining component necessity, optimal combination, sequential appropriateness, and the necessary boundaries between independent self-care and professional supervision.

## 5. Conclusions

This study developed a consensus-based TCM-L framework for insomnia through a four-round modified Delphi process. The finalized framework comprises six domains and 49 items, along with provisional item-importance rankings and a sequence for domain delivery. Rather than serving as a direct clinical protocol or treatment recommendation, this framework provides a structured foundational basis for insomnia self-management education. While further operational development, safety evaluation, and clinical testing are required before broader implementation, these findings may inform the standardization of TCM-based health education, curriculum design, and the development of future interventions.

## Figures and Tables

**Table 1:** General information about the experts (*n* = 18).

Category	Frequency [*n* (%)]
Professional qualification	
Registered Chinese medicine practitioner (HK)	18 (100%)
Gender	
Male	8 (44.4%)
Female	10 (55.6%)
Age (years)	
≤30	2 (11.1%)
31–40	8 (44.4%)
41–50	6 (33.3%)
>50	2 (11.1%)
Highest education completed	
Bachelor’s degree	1 (5.6%)
Master’s degree	11 (61.1%)
Doctoral degree (e.g., PhD)	6 (33.3%)
Years of working experience (years)	
<10	6 (33.3%)
10–19	6 (33.3%)
≥20	6 (33.3%)
Work setting/Clinical type	
Private practice	6 (33.3%)
HA-NGO-University tripartite CM clinics *	5 (27.8%)
University/Academic institution	6 (33.3%)
NGO	1 (5.5%)

Note: * HA-NGO-University tripartite CM clinics refer to the Chinese Medicine Clinics cum Training and Research Centers in Hong Kong; NGO = non-governmental organization; CM = Chinese medicine.

**Table 2:** Expert authority coefficients across the Delphi rounds.

Round	Judgment Basis (*Ca*)	Familiarity Coefficient (*Cs*)	Authority Coefficient (*Cr*)
Round 1	0.911	0.856	0.883
Round 2	0.833	0.894	0.864
Round 3	0.900	0.867	0.883
Round 4	0.872	0.867	0.869

**Table 3:** Degree of Expert Coordination Across Delphi Rounds.

Round	Evaluation Target	Items (*n*)	Kendall’s *W*	*X* ^2^	*p*-Value
Round 1 (Scoring)	Domains	10	0.353	57.274	<0.001
	Items	82	0.318	463.074	<0.001
Round 2 (Scoring)	Domains	6	0.372	33.516	<0.001
	Items	51	0.233	209.250	<0.001
Round 3 (Scoring)	Domains	6	0.194	17.419	0.004
	Items	55	0.180	178.671	<0.001
Round 4 (Ranking)	Importance of domains	6	0.717	64.54	<0.001
	Items in domain 1	14	0.249	58.36	<0.001
	Items in domain 2	7	0.503	54.33	<0.001
	Items in domain 3	8	0.392	49.33	<0.001
	Items in domain 4	8	0.300	35.75	<0.001
	Items in domain 5	8	0.273	34.389	<0.001
	Items in domain 6	4	0.272	14.667	0.002

**Table 4:** Summary of item and domain modifications across Delphi consultation rounds.

Round	Total		Added	Merged	Deleted	Revised
	Domain	Item				
Round 1	10	82	+12	7 items merged into 3 items (−4)	−4 domains; −39 items (4 in domain 2; 3 in domain 3; 1 in domain 5; 23 in domain 7, 5 in domain 8; 3 in domain 10	3
Round 2	6	51	+11	2 items merged into 1 (−1)	−6 items (1 in domain 2; 2 in domain 4; 3 in domain 6)	8
Round 3	6	55	+1	4 items merged into 1 (−3) Sub-items of 2.2 and 2.3 merged (−2)	−2 items (1 in domain 2; 1 in domain 6)	1
Round 4	6	49	-	-	-	-

**Table 5:** Final TCM-L content framework.

Content	Importance Ranking
Rank 1 (Domain 1: Introduction to TCM-L framework)	
TCM definition of insomnia (dissatisfaction with sleep quantity/quality without physical disease)	1
Etiology of insomnia (key factors including dietary irregularities, emotional distress, and imbalance between work and rest)	2
TCM sleep theories (exploring how sleep is regulated by the circadian balance of yin-yang, internal energy circulation, and the governance of the mind)	3
Theoretical basis of TCM health preservation (principles of *yin-yang*, five elements, and the “unity of nature and humanity”)	4
Definition of TCM health preservation (traditional theories and methods for body–mind cultivation and disease prevention)	5
Five-dimensional content of TCM health preservation (sleep and wake routines, diet, emotion, mind–body exercise, and meridian)	6
Pathogenesis of insomnia (key mechanism involving heart–spirit instability, related to the heart, liver, spleen, and kidney)	7
Relationship between *Zang-fu* organs and insomnia (exploring how various etiologies affect different organs)	8
Self-diagnosis of TCM body constitution types of insomnia (common constitutions include *qi* deficiency, *yin* deficiency); individuals with different constitutions will exhibit different clinical manifestations when experiencing insomnia)	9
TCM non-pharmacological therapies and multidimensional interventions (e.g., acupoint stimulation, external herbal treatments, moxibustion, massage, emotional regulation, and exercise), integrating physiological, psychological, and lifestyle adjustments)	10
TCM treatment principles (with regulation of *Zang-fu*, harmonization of *qi* and blood, and calming of the mind)	10
Safety, target population, and precautions (emphasizing natural and gentle regulation with high safety; suitable for most patients with insomnia seeking non-pharmacological methods; requires individualized adjustments and professional guidance for severe cases or special groups like pregnant women)	12
Group discussion on prevention methods (maintaining emotional stability, regular lifestyle, and healthy sleep hygiene)	13
TCM therapies for specific pathogenesis (applying targeted techniques based on syndrome types; e.g., using acupoint massage and dietary therapy to nourish the heart and spleen for patients with heart–spleen deficiency)	14
Rank 2 (Domain 4: Sleep–wake routines)	
Theory of sleep–wake routines (harmonizing with nature, balancing work and rest, and optimizing the living environment)	1
Definition of daily living health preservation (regulating daily routines to align with natural and physiological rhythms)	2
Methods for judging sleep quality (assessing sleep onset speed, frequency of waking, depth of sleep, and morning alertness)	3
Formulation of personal sleep plans (developing a scientific daily sleep schedule tailored to individual needs)	4
The “Midday-Midnight Circadian *Qi* Flow” (*Zi-Wu-Liu-Zhu*) [sleeping during *Zi* (23:00–01:00) and napping during *Wu* (11:00–13:00, <1 h)]	5
TCM herbal aromatherapy for insomnia tailored to various body types (with hands-on sachet making)	6
Taking a warm bath or soaking feet before bedtime	7
Sleep environment and selection of bedding (ensuring a quiet environment with comfortable pillows, mattresses, and sleepwear)	7
Rank 3 (Domain 3: Dietary regulation)	
TCM basic theories of dietary regulation (including the “four natures” of food: cold, cool, warm, and hot; and the “five flavors”: sour, bitter, sweet, pungent, and salty)	1
Principles of dietary regulation (individualized dietary strategies, moderation, and dietary taboos)	2
Functions of dietary regulation (nourishing the body, delaying aging, and strengthening resistance against pathogenic factors)	3
TCM definition of dietary regulation (selecting and harmonizing foods based on TCM theories to balance *yin-yang* and prevent disease)	4
Dietary strategies for different constitutions	5
Selection of dietary ingredients [proper use of five grains (staples), vegetables, fruits, and meats; appropriate use of seasonings]	6
Health-preserving herbal teas (using herbal tea formulas, such as wolfberry and chrysanthemum, to clear fire and nourish yin)	7
Precautions for dietary health preservation	8
Rank 4 (Domain 2: Emotional regulation)	
Definition of emotional regulation (cultivating virtue and spirit to enhance psychological well-being based on TCM theories)	1
Self-regulation (regulating and managing emotions, preventing extreme emotional reactions, and maintaining mental stability can help improve sleep)	2
Catharsis method (releasing suppressed emotions through communication or healthy venting)	3
*Qi* regulation method (harmonizing *Zang-fu* functions and “spirit” via traditional exercises, e.g., body-breath-mind adjustment)	4
Avoiding the use of electronic products before bed (laptops or smartphones)	5
Avoiding extreme emotions before bed (preventing “heart–spirit” disturbance caused by intense emotional fluctuations)	6
Transforming the mindset and overcoming one emotion with another	6
Rank 5 (Tied) (Domain 6: Acupoint massage)	
Practical in-class training of key acupoints [hands-on practice for easily operable points: *Baihui* (GV20), *Shenmen* (HT7), *Neiguan* (PC6), and *Yintang* (GV29), operational precautions and safety guidelines (managing pressure/timing; special advice for pregnant women or those with skin conditions]	1
Definition and functions of acupoint massage	1
Hand-on-navel and *Qi*-sinking-to-*Dantian*	3
*Yongquan* massage: *yin* restoration and sleep quality	4
Rank 5 (Tied) (Domain 5: Mind–body exercise)	
Characteristics and functions (harmonizing *qi* and blood and improving *Zang-fu* functions through dynamic/static balance)	1
Definition of mind–body exercise (integrating body movements and breathing to achieve “unity of body and spirit”)	2
Key essentials (exercise and regulation of body, *qi*, and spirit)	2
Principles of practice (moderation, individualization, and persistent, step-by-step progress)	4
Leading participants to practice *Baduanjin* in class	5
Common exercise practices (e.g., *Tai Chi* and *Baduanjin*) introduced via video	6
Pre-sleep relaxation exercises (gentle movements like yoga to calm the mind)	6
Precautions and individualized suggestions (adjusting intensity based on constitution and avoiding vigorous exercise before bed)	8

Note: Italicized terms indicate Chinese pinyin.

## Data Availability

The datasets used and/or analyzed in this study are available from the corresponding author upon reasonable request.

## Supplementary Materials

The following supporting information can be downloaded at: https://www.mdpi.com/article/10.3390/healthcare14182907/s1, Figure S1. Studies included in the systematic review (*n* = 14); Table S1. Results of the TCM-based lifestyle (TCM-L) content framework for insomnia: Delphi round 1; Table S2. Results of the TCM-based lifestyle (TCM-L) content framework for insomnia: Delphi round 2; Table S3. Results of the TCM-based lifestyle (TCM-L) content framework for insomnia: Delphi round 3; Table S4. Results of Delphi round 4: Importance ranking of items within each domain; Table S5. Results of Delphi round 4: Recommended curriculum sequence of the six domains.
