# Supplementary material for: Developing a Traditional Chinese Medicine-Based Lifestyle Content Framework for Insomnia: A Modified Delphi Study
Source: Healthcare (Basel). 2026 Sep 8;14(18):2907. doi: 10.3390/healthcare14182907 (PMC13606197; doi:10.3390/healthcare14182907)
Supplement: Supplementary file 1 [file healthcare-14-02907-s001.zip › healthcare-4461526-supplementary.pdf]

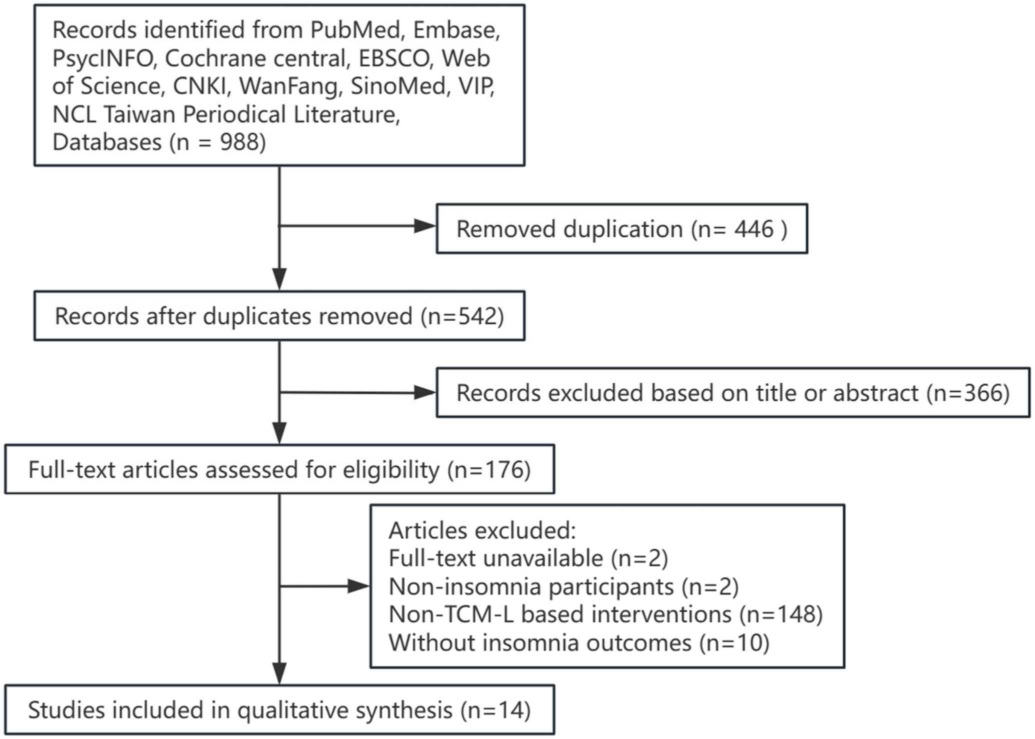

Figure S1. Studies included in the systematic review (n = 14)

Table S1. Results of the TCM-based lifestyle (TCM-L) content framework for insomnia: Delphi round 1

Content	Mean	SD [a]	CV [b]	CLA (%) [c]	Consensus States	Action Taken	Added
**Domain 1. Introduction to TCM-L framework**	4.61	0.50	0.11	100.00%			
1.1 TCM definition of insomnia (dissatisfaction with sleep quantity/quality without physical disease)	4.67	0.49	0.10	100.00%			2 items: "TCM body constitution types of insomnia" "Safety, target population,
1.2 Etiology of insomnia (key factors including dietary irregularities, emotional distress, and imbalance between work and rest)	4.78	0.55	0.11	94.44%			
1.3 Pathogenesis of insomnia (key mechanism involving heart-spirit instability, related to the heart, liver, spleen, and kidney)	4.44	0.70	0.16	88.89%			
1.4 TCM treatment methods (include Chinese herbal medicine, acupuncture, moxibustion, massage, and *qigong*)	4.67	0.69	0.15	88.89%		Merged (with 1.7)	

1.5 TCM treatment principles (with the regulation of the internal organs, harmonization of *qi* and blood, and calming of the mind)	4.61	0.61	0.13	94.44%			and precautions"
1.6 Multidimensional TCM interventions (holistic conditioning integrating physiological, psychological, and lifestyle adjustments)	4.50	0.86	0.19	88.89%			
1.7 Non-pharmacological TCM therapies (e.g., acupoint stimulation, external herbal treatments, emotional regulation, and exercise)	4.67	0.77	0.16	94.44%		Merged (with 1.4)	
1.8 Preventive care and health maintenance for insomnia (maintaining emotional stability, regular lifestyle, and healthy sleep hygiene)	4.61	0.78	0.17	94.44%			
1.9 Definition of TCM health preservation (traditional theories and methods for body-mind cultivation and disease prevention)	4.33	0.77	0.18	94.44%			
1.10 Theoretical basis of TCM health preservation (principles of *yin-yang*, *Zang-fu* organs, and the "unity of nature and humanity")	4.24	1.03	0.24	83.33%			
1.11 Nine dimensions of TCM health preservation (emotions, social life, dietary, sleep and wake routines, sexual, cultivating hobbies, bathing, mind-body exercise, and meridian health preservation)	4.33	0.69	0.16	88.89%			
**Domain 2. Emotional regulation**	4.61	0.50	0.11	100.00%			
2.1 Definition of emotional regulation (cultivating virtue and spirit to enhance psychological well-being based on TCM theories)	4.28	0.57	0.13	94.44%			1 item: "Self-regulation"
2.2 Essentials of pre-sleep emotional regulation	4.56	0.62	0.14	94.44%			
2.2.1 Avoid extreme emotions (preventing "heart-spirit" disturbance caused by intense emotional fluctuations)	4.56	0.62	0.14	94.44%			
2.2.2 Cease overthinking (reducing excessive rumination that "injures the spleen" and disturbs mental tranquility)	4.50	0.79	0.17	94.44%		Revised (halting overthinking is often unrealistic for insomnia patients, so this item was revised to "transform the mindset".)	
2.2.3 Meditation for calming the mind (utilizing mindfulness or focused stillness to stabilize inner peace)	4.33	0.69	0.16	88.89%			
2.3 Methods of emotional regulation	4.61	0.61	0.13	94.44%			
2.3.1 Distraction method (shifting focus through hobbies such as arts, sports to resolve	4.44	0.62	0.14	94.44%			

negative emotions)							
2.3.2 Suggestion method (using subtle gestures or expressions to influence mental and behavioral states)	3.33	0.91	0.27	38.89%	Failed	Deleted	
2.3.3 Guidance method (resolving negative emotions through persuasive dialogue, encouragement, and reassurance)	4.17	0.92	0.22	77.78%	Failed	Deleted	
2.3.4 Restraint method (self-regulation and moderation of the "seven emotions" to maintain psychological balance)	4.17	1.04	0.25	83.33%	Failed	Deleted	
2.3.5 Catharsis method (releasing suppressed emotions through communication or healthy venting)	4.22	1.06	0.24	83.33%			
2.3.6 *Qi* regulation method (harmonizing *Zang-fu* functions and "spirit" via traditional exercises, e.g., body-breath-mind adjustment)	4.61	0.61	0.13	94.44%			
2.3.7 Emotional counterbalance method (utilizing the five elements theory to restrain one emotion with another, e.g., joy overcomes grief)	3.94	1.00	0.25	77.78%	Failed	Deleted	
**Domain 3. Social adaptability**	4.22	0.81	0.19	77.78%	Failed	Deleted	
3.1 Definition of social adaptability (utilizing social environments to improve interpersonal relationships and emotional well-being)	4.17	0.79	0.19	77.78%	Failed	Deleted	
3.2 Health-preserving functions (Alleviating loneliness, enhancing warmth and happiness, and maintaining psychological balance)	4.11	0.76	0.18	77.78%	Failed	Deleted	
3.3 Principles and measures (Principles: honesty, respect, and tolerance. Measures: empathy, smiling, and polite communication)	4.11	0.76	0.18	77.78%	Failed	Deleted	
**Domain 4. Dietary regulation**	4.83	0.38	0.08	100.00%			
4.1 Dietary principles (balanced nutrition, individualized meals, and moderation in food/drink intake)	4.78	0.43	0.09	100.00%			6 items: "TCM definition, theories and functions of dietary health preservation", "Balanced diet,
4.2 Selection of food ingredients (proper use of five grains, vegetables, fruits, and meats; appropriate use of seasonings)	4.61	0.61	0.13	94.44%			
4.3 Syndrome differentiation-based diet (medicinal food based on insomnia types)	4.39	0.78	0.18	83.33%			
4.4 Tea and soup therapy (customized prescriptions (e.g., sour date seed tea, *Ganmai Hongzao* decoction) based on body types)	4.61	0.61	0.13	94.44%		Revised (preparing herbal tea is more straightforward and less time-consuming than decocting soup)	

4.5 Pre-sleep sleep-promoting foods (consumption of calming foods like walnuts, honey, and lily bulbs (*Baihe*) or medicinal diets)	4.22	1.22	0.29	77.78%	Failed	Deleted	individualized dietary strategies, moderation and dietary taboos”
**Domain 5. Sleep-wake routines**	4.61	0.50	0.11	100.00%			
5.1 Definition of sleep-wake routines (regulating daily routines to align with natural and physiological rhythms)	4.72	0.46	0.10	100.00%			
5.2 Theories of daily living (harmonizing with nature, balancing work and rest, and optimizing the living environment)	4.72	0.46	0.10	100.00%			
5.3 The “midday-midnight circadian *qi* flow” (*Zi-Wu-Liu-Zhu*) (sleeping during zi (23:00-01:00) and napping during wu (11:00-13:00, <1 hour))	4.56	0.62	0.14	94.44%			
5.4 Bedtime regulation methods (e.g., foot baths, relaxing the mind, light stretching)	4.22	0.88	0.21	72.22%	Failed	Merged (with 5.6)	
5.5 Sleeping postures and orientations (optimizing physical alignment and direction during sleep for better energy flow)	3.39	0.98	0.29	38.89%	Failed	Revised (specify the operational posture)	
5.6 Sleep environment and selection of bedding (ensuring a quiet environment with comfortable pillows, mattresses, and sleepwear)	4.61	0.61	0.13	94.44%			
5.7 Sleep taboos and precautions (e.g., avoiding worry and anger, avoiding extreme hunger or fullness)	4.05	0.18	0.72	77.78%	Failed	Merged (with 5.4)	
5.8 Methods for judging sleep quality (assessing sleep onset speed, frequency of waking, depth of sleep, and morning alertness)	4.33	0.91	0.21	83.33%			
5.9 Formulation of personal sleep plans (developing a scientific daily sleep schedule tailored to individual needs)	4.29	0.69	0.16	83.33%			
**Domain 6. Sexual regulation**	3.78	0.94	0.25	66.67%	Failed	Deleted	
6.1 Definition of sexual regulation (sexual health care based on physiological laws and psychological characteristics)	3.89	0.96	0.25	72.22%	Failed	Deleted	
6.2 Functions of sexual health preservation (soothing emotions, regulating *qi*, and strengthening the body/intelligence)	3.83	0.92	0.24	72.22%	Failed	Deleted	
6.3 Practical measures (maintaining moderate frequency, e.g., 2-3 times/week for adults; once/month for the elderly)	3.78	0.88	0.23	72.22%	Failed	Deleted	
6.4 Sexual taboos (specific restrictions to avoid injury to essence and spirit)	3.89	1.02	0.26	66.67%	Failed	Deleted	

**Domain 7. Cultivating hobbies**	3.78	0.88	0.23	50.00%	Failed	Deleted	
7.1 Definition of cultivating hobbies (cultivating refined interests and hobbies to nourish the body and mind)	3.89	0.83	0.21	61.11%	Failed	Deleted	
7.2 Importance of daily hobbies (preventing nighttime insomnia by structuring daytime activities and interests)	3.89	0.83	0.21	61.11%	Failed	Deleted	
7.3 Music health preservation (mechanisms and essentials of using music for health)	3.72	1.07	0.29	55.56%	Failed	Deleted	
7.3.1 In-class practice: domestic melodies (appreciation of Chinese classical music like *Gaoshan Liushui*)	3.61	1.09	0.30	55.56%	Failed	Deleted	
7.3.2 In-class practice: international melodies (appreciation of Western classical music, e.g., the Blue Danube)	3.67	1.14	0.31	55.56%	Failed	Deleted	
7.3.3 Pre-sleep calming methods (using low-volume, soothing music (e.g., five-element music) to aid sleep)	3.83	1.10	0.29	61.11%	Failed	Deleted	
7.3.4 Precautions for music therapy (selecting appropriate timing, volume, and environment)	3.89	1.02	0.26	72.22%	Failed	Deleted	
7.4 Chess/Strategy games (enhancing mental tranquility and internal organ function through concentration)	3.28	1.02	0.31	38.89%	Failed	Deleted	
7.5 Calligraphy and painting (regulating *qi*, clearing meridians, and calming the mind)	3.56	1.04	0.29	55.56%	Failed	Deleted	
7.5.1 In-class practice: appreciation of calligraphy/painting works	3.44	0.98	0.29	50.00%	Failed	Deleted	
7.6 Reading health preservation (improving temperament and adjusting emotions through aesthetic reading)	3.61	1.04	0.29	61.11%	Failed	Deleted	
7.6.1 In-class practice: reading health-preserving essays	3.39	0.98	0.29	44.44%	Failed	Deleted	
7.7 Fishing (strengthening the body and calming the spirit in a natural setting)	3.17	1.04	0.33	33.33%	Failed	Deleted	
7.8 Flower and bird appreciation (regulating emotions and smoothing *qi* flow through nature)	3.44	1.04	0.30	44.44%	Failed	Deleted	
7.9 Travel (enjoying nature through "suburban outings" while ensuring safety and rest)	3.78	0.88	0.23	61.11%	Failed	Deleted	
7.10 Tea tasting (refreshing the mind and fostering character through tea culture)	3.67	1.03	0.28	55.56%	Failed	Deleted	
7.11 Collecting (gaining mental fulfillment and cultural knowledge through collections)	3.28	1.07	0.33	33.33%	Failed	Deleted	
7.12 Aromatherapy (using essential oils (e.g., lavender, rose) to soothe the spirit and aid sleep)	3.72	0.75	0.20	55.56%	Failed	Deleted	
**Domain 8. Bathing therapy**	3.67	0.84	0.23	55.56%	Failed	Deleted	
8.1 Definition of bathing therapy (utilizing water, herbs, or natural media to strengthen the body and regulate *qi*)	3.56	0.86	0.24	55.56%	Failed	Deleted	
8.2 Water bath (physical and chemical effects of hot, cold, steam, or spring baths)	3.67	0.91	0.25	61.11%	Failed	Deleted	
8.3 Medicated bath (using sleep-promoting herbal formulas (e.g., *Yejiaoteng*) for immersion	4.00	0.69	0.17	77.78%	Failed	Deleted	

or steaming)							
8.4 Other methods: mud, sun, air, and seawater baths for diverse health benefits.	3.50	0.92	0.26	44.44%	Failed	Deleted	
8.5 Pre-sleep foot bath methods (stimulating acupoints (e.g., *Yongquan*) via warm herbal decoctions to calm the heart)	4.11	0.76	0.18	77.78%	Failed	Deleted	
**Domain 9. Mind–body exercise**	4.33	0.59	0.14	94.44%			
9.1 Definition of mind–body exercise (integrating body movements and breathing to achieve "unity of body and spirit")	4.33	0.59	0.14	94.44%			
9.2 Characteristics and functions (harmonizing *qi* and blood, and improving *Zang-fu* functions through dynamic/static balance)	4.24	0.66	0.16	83.33%			
9.3 Key essentials (the exercise and regulation of body, *qi*, and spirit)	4.22	0.65	0.15	88.89%			
9.4 Principles of practice (moderation, individualization, and persistent, step-by-step progress)	4.17	0.62	0.15	88.89%			
9.5 Common exercise practices (e.g., *Tai Chi* and *Baduanjin*)	4.33	0.69	0.16	88.89%			
9.6 Pre-sleep relaxation exercises (gentle movements like yoga to calm the mind)	4.35	0.61	0.14	88.89%			
9.7 Precautions and individualized suggestions (adjusting intensity based on constitution and avoiding vigorous exercise before bed)	4.28	0.67	0.16	88.89%			
**Domain 10. Meridian regulation**	4.33	0.77	0.18	83.33%		Revised (only 3 items remained)	
10.1 Specific acupoint conditioning based on insomnia types (selecting points based on syndrome, e.g., *Qimen* and *Taichong* for liver; *Zusanli* for spleen/stomach)	4.33	0.84	0.19	77.78%	Failed	Deleted	3 items: "TCM herbal foot bath" "TCM aromatherapy nursing for insomnia" "TCM five-tone therapy for insomnia"
10.2 Auxiliary methods for meridian dredging [techniques such as scraping (*gua sha*), cupping, and moxibustion to clear heat and dampness]	4.22	0.81	0.19	77.78%	Failed	Deleted	
10.3 Practical in-class training of key acupoints (hands-on practice for easily operable points: *Baihui* (GV20), *Shenmen* (HT7), *Neiguan* (PC6), and *Yintang* (GV29))	4.44	0.78	0.18	83.33%		Merged (with 10.5 and 10.7)	
10.4 Ear seed pressing method (applying seeds to ear points (heart, *Shenmen*, subcortex, and sympathetic) to regulate the nervous system)	4.28	0.96	0.22	77.78%	Failed	Deleted	
10.5 Self-massage techniques for sleep promotion (instruction on rhythmic massage sequences and pressure techniques for home use)	4.44	0.78	0.18	83.33%		Merged (with 10.3 and 10.7)	
10.6 Kneading terminal segments of fingers and toes (rhythmic pinching and kneading from the small toe to the big toe to regulate *qi* flow)	4.06	0.94	0.23	61.11%	Failed	Deleted	
10.7 Operational precautions and safety guidelines (managing pressure/timing; special advice for pregnant women or those with skin conditions)	4.56	0.70	0.15	88.89%		Merged (with 10.3 and 10.7)	

a SD: Standard Deviation;

b CV: Coefficient of Variation;

c CLA: Consensus Level of Agreement;

Consensus Status: “Failed” indicates the item did not simultaneously meet the pre-defined thresholds (M $\geqslant$ 4.0, CV < 0.25, and CLA $\geqslant$ 80%);

Action Taken: Includes items that were revised, merged, or deleted during the Delphi rounds.

Table S2. Results of the TCM-based lifestyle (TCM-L) content framework for insomnia: Delphi round 2

TCM-L intervention framework	Mean	SD [a]	CV [b]	CLA (%) [c]	Consensus Status	Action Taken	Add
**Domain 1. Introduction to TCM-L framework**	4.78	0.43	0.09	100.00%			
1.1 TCM definition of insomnia (dissatisfaction with sleep quantity/quality without physical disease)	4.83	0.38	0.08	100.00%			3 items: "TCM sleep theories", "relationship between the *Zang-fu* organs and insomnia", "TCM therapies for specific pathogenesis"
1.2 Etiology of insomnia (key factors including dietary irregularities, emotional distress, and imbalance between work and rest)	4.72	0.57	0.12	94.44%			
1.3 Pathogenesis of insomnia (key mechanism involving heart–spirit instability, related to the heart, liver, spleen, and kidney)	4.61	0.70	0.15	88.89%			
1.4 TCM treatment principles (with the regulation of the *Zang-fu*, harmonization of *qi* and blood, and calming of the mind)	4.67	0.59	0.13	94.44%			
1.5 TCM treatment methods and non-pharmacological therapies (e.g., acupoint stimulation, external herbal treatments, moxibustion, massage, emotional regulation, and exercise)	4.83	0.38	0.08	100.00%		Merged (with 1.6)	
1.6 Multidimensional interventions (holistic conditioning integrating physiological, psychological, and lifestyle adjustments)	4.83	0.38	0.08	100.00%		Merged (with 1.5)	
1.7 Preventive care and health maintenance for insomnia (maintaining emotional stability, regular lifestyle, and healthy sleep hygiene)	4.83	0.38	0.08	100.00%		Revised (adding group discussion)	
1.8 TCM body constitution types of insomnia (common constitutions include *qi* deficiency, yin deficiency, etc.; Individuals with different constitutions will exhibit different clinical manifestations when experiencing insomnia)	4.39	0.85	0.19	77.78%	Failed	Revised (adding self-diagnosis of TCM body constitution)	
1.9 Definition of TCM health preservation (traditional theories and methods for body-mind cultivation and disease prevention)	4.39	0.70	0.16	88.89%			
1.10 Theoretical basis of TCM health preservation (based on TCM theory, covering core concepts such as life, longevity, and harmony, and principles like prevention and holistic cultivation)	4.00	0.91	0.23	72.22%	Failed	Revised	

1.11 Nine dimensions of TCM health preservation (emotion, social, dietary, sleep and wake routines, sexual, cultivating hobbies, bathing, mind-body exercise, and meridian health preservation)	3.78	1.06	0.28	55.56%	Failed	Revised (only the domains to be taught are retained)	
1.12 Safety, target population, and precautions (emphasizing natural and gentle regulation with high safety; suitable for most insomnia patients seeking non-pharmacological methods; requires individualized adjustments and professional guidance for severe cases or special groups like pregnant women)	4.28	0.75	0.18	83.33%			
**Domain 2. Emotional regulation**	4.61	0.50	0.11	100.00%			
2.1 Definition of emotional regulation (cultivating virtue and spirit to enhance psychological well-being based on TCM theories)	4.44	0.78	0.18	83.33%			1 item: Avoid using electronic products before bed
2.2 Essentials of pre-sleep emotional regulation	4.50	0.62	0.14	94.44%			
2.2.1 Avoiding extreme emotions (preventing “heart-spirit” disturbance caused by intense emotional fluctuations)	4.61	0.61	0.13	94.44%			
2.2.2 Transforming the mindset (replacing excessive rumination with mental relaxation to calm the mind and facilitate sleep onset)	4.56	0.62	0.14	94.44%		Revised (transforming the mindset and overcoming one emotion with another)	
2.2.3 Meditation for calming the mind (utilizing mindfulness or focused stillness to stabilize inner peace)	3.94	1.21	0.31	66.67%	Failed	Deleted	
2.3 Methods of emotional regulation	4.67	0.49	0.10	100.00%			
2.3.1 Distraction method (shifting focus through hobbies such as arts, sports, to resolve negative emotions)	4.22	1.00	0.24	88.89%			
2.3.2 Self-regulation (regulating and managing emotions, preventing excessive emotional reactions, and maintaining psychological balance as beneficial for improving sleep)	4.50	0.51	0.11	100.00%			

2.3.3 Catharsis method (releasing suppressed emotions through communication or healthy venting)	4.44	0.70	0.16	88.89%			
2.3.4 *Qi* regulation method (harmonizing *Zang-fu* functions and "spirit" via traditional exercises, e.g., body-breath-mind adjustment)	4.33	0.69	0.16	88.89%			
**Domain 3. Dietary regulation**	4.89	0.32	0.07	100.00%			
3.1 TCM definition of dietary regulation (selecting and harmonizing foods based on TCM theories to balance *Yin-Yang* and prevent disease)	4.72	0.57	0.12	94.44%			1 item: Precautions for dietary regulation
3.2 TCM basic theories of dietary regulation (including the "four natures" of food: cold, cool, warm, hot; and the "five flavors": sour, bitter, sweet, pungent, and salty)	4.67	0.59	0.13	94.44%			
3.3 Functions of dietary regulation (nourishing the body, delaying aging, and strengthening resistance against pathogenic factors)	4.50	0.79	0.17	83.33%			
3.4 Principles of dietary regulation	4.67	0.59	0.13	94.44%			
3.4.1 Balanced diet with appropriate combination of foods (emphasis on diverse food groups and rational nutritional structure, for example, food guide pagoda)	4.39	0.70	0.16	88.89%			
3.4.2 Individualized dietary strategies (tailoring diets according to personal constitution, seasonal changes, and geographical environment)	4.78	0.55	0.11	94.44%			
3.4.3 Moderation and dietary taboos (regulating meal timing and quantity, while avoiding irritants like caffeine or heavy food before bed)	4.67	0.49	0.10	100.00%			
3.5 Selection of dietary ingredients (proper use of five grains, vegetables, fruits, and meats; appropriate use of seasonings)	4.28	0.75	0.18	83.33%			
3.6 Syndrome differentiation-based diet (medicinal food based on insomnia types)	4.78	0.43	0.09	100.00%			
3.7 Health-preserving herbal teas (using herbal tea formulas, such as wolfberry and chrysanthemum, to clear fire and nourish yin)	4.56	0.62	0.14	94.44%			
**Domain 4. Sleep-wake routines**	4.39	0.61	0.14	94.44%			
4.1 Definition of sleep-wake routines (regulating daily routines to align with natural and physiological rhythms)	4.50	0.51	0.11	100.00%			2 items: "TCM herbal aromatherapy for insomnia tailored to various body types"
4.2 Theories of daily living (harmonizing with nature, balancing work and rest, and optimizing the living environment)	4.50	0.51	0.11	100.00%			
4.3 The "midday-midnight circadian *qi* flow" (*Zi-Wu-Liu-Zhu*) [sleeping during zi (23:00-01:00) and napping during wu (11:00-13:00, <1 hour)]	4.28	0.89	0.21	72.22%	Failed	Revised (even if a perfect alignment	

						with nature isn't possible, keeping a regular sleep routine is essential and should be maintained as much as possible)	"Taking a warm bath or soaking feet before bedtime"
4.4 Bedtime regulation methods and precautions (e.g., foot baths, relaxing the mind, light stretching, avoiding worry and anger; speaking less; avoiding extreme hunger or fullness)	4.26	0.73	0.18	77.78%	Failed	Deleted	
4.5 Sleeping posture and orientations (e.g., right lateral position with a slightly curved "bow-like" posture)	3.72	0.89	0.24	55.56%	Failed	Deleted	
4.6 Sleep environment and selection of bedding (ensuring a quiet environment with comfortable pillows, mattresses, and sleepwear)	4.17	0.86	0.21	83.33%			
4.7 Methods for judging sleep quality (assessing sleep onset speed, frequency of waking, depth of sleep, and morning alertness)	4.67	0.49	0.10	100.00%			
4.8 Formulation of personal sleep plans (developing a scientific daily sleep schedule tailored to individual needs)	4.56	0.62	0.14	94.44%			
**Domain 5. Mind–body exercise**	4.33	0.59	0.14	94.44%			
5.1 Definition of mind–body exercise (integrating body movements and breathing to achieve "unity of body and spirit")	4.39	0.70	0.16	88.89%			1 item: Lead the participants to practice *Baduanjin* in the class.[v]
5.2 Characteristics and functions (harmonizing *qi* and blood, and improving *Zang-fu* functions through dynamic/static balance)	4.39	0.50	0.11	100.00%			
5.3 Key essentials (the exercise and regulation of body, *qi,* and spirit)	4.28	0.67	0.16	88.89%			
5.4 Principles of practice (moderation, individualization, and persistent, step-by-step progress)	4.33	0.69	0.16	88.89%			
5.5 Common exercise practices (e,g. *Taichi* and *Baduanjin*)	4.44	0.51	0.12	100.00%		Revised	

						(this can be delivered via video instruction.)	
5.6 Pre-sleep relaxation exercises (gentle movements like Yoga to calm the mind)	4.28	0.75	0.18	83.33%			
5.7 Precautions and individualized suggestions (adjusting intensity based on constitution and avoiding vigorous exercise before bed)	4.50	0.62	0.14	94.44%			
**Domain 6. Acupoint massage**	4.00	0.77	0.19	72.22%	Failed	Revised （emphasizing self-massage）	Domain 6: Self-meridian acupoint massage
6.1 TCM herbal foot bath (e.g., use of *Suanzaoren, Baiziren, Yuanzhi* for mind-calming and blood circulation via warm soaking and transdermal absorption)	3.89	0.76	0.20	66.67%	Failed	Deleted	3 items: "Bedtime foot shaking": "hand-on-navel and *qi*-sinking-to-dantian" "*yongquan* massage"
6.2 TCM aromatherapy nursing for insomnia (e.g., use of calming and sedative herbs such as *Suanzaoren*, *Wuweizi, Benzoin, Gansong*, etc)	3.61	1.14	0.32	66.67%	Failed	Deleted	
6.3 TCM five-tone therapy for insomnia (The five tones—*Gong, Shang, Jue, Zhi, Yu*—resonating with the spleen, lung, liver, heart, and kidney)	3.39	1.09	0.32	44.44%	Failed	Deleted	
6.4 Practical in-class training of key acupoints [hands-on practice for easily operable points: *Baihui* (GV20), *Shenmen* (HT7), *Neiguan* (PC6), and *Yintang* (GV29), operational precautions and safety guidelines (managing pressure/timing; special advice for pregnant women or those with skin conditions)	4.72	0.46	0.10	100.00%			

a SD: Standard Deviation;

b CV: Coefficient of Variation;

c CLA: Consensus Level of Agreement;

Consensus Status: "Failed" indicates the item did not simultaneously meet the pre-defined thresholds (M ⩾ 4.0, CV < 0.25, and CLA ⩾ 80%);

Action Taken: Includes items that were revised, merged, or deleted during the Delphi rounds.

Table S3. Results of the TCM-based lifestyle (TCM-L) content framework for insomnia: Delphi round 3

Content	Mean	SD [a]	CV [b]	CLA (%) [c]	Consensus Status	Action Taken	Add
**Domain 1. Introduction to TCM-L framework**	4.89	0.32	0.07	100.00%			
1.1 TCM definition of insomnia (dissatisfaction with sleep quantity/quality without physical disease)	4.61	0.78	0.17	94.44%			
1.2 TCM sleep theories (exploring how sleep is regulated by the circadian balance of yin-yang, internal energy circulation, and the governance of the mind) [v]	4.44	0.62	0.14	94.44%			
1.3 Etiology of insomnia (key factors including dietary irregularities, emotional distress, and imbalance between work and rest)	4.78	0.55	0.11	94.44%			
1.4 Pathogenesis of insomnia (key mechanism involving heart–spirit instability, related to the heart, liver, spleen, and kidney)	4.56	0.62	0.14	94.44%			
1.5 TCM treatment principles (with the regulation of the *Zang-fu,* harmonization of *qi* and blood, and calming of the mind)	4.61	0.61	0.13	94.44%			
1.6 TCM non-pharmacological therapies and multidimensional interventions (e.g., acupoint stimulation, external herbal treatments, moxibustion, massage, emotional regulation, and exercise. holistic conditioning integrating physiological, psychological, and lifestyle adjustments)	4.61	0.50	0.11	100.00%			
1.7 TCM therapies for specific pathogenesis (applying targeted techniques based on syndrome types; e.g., using acupoint massage and dietary therapy to nourish the heart and spleen for patients with heart-spleen deficiency)	4.56	0.51	0.11	100.00%			
1.8 Group discussion on prevention methods (maintaining emotional stability, regular lifestyle, and healthy sleep hygiene)	4.56	0.62	0.14	94.44%			
1.9 Self-diagnosis of TCM body constitution types of insomnia (common constitutions include *qi* deficiency, yin deficiency, etc. Individuals with different constitutions will exhibit different clinical manifestations when experiencing insomnia)	4.39	0.78	0.18	83.33%			
1.10 Relationship between *Zang-fu* organs and insomnia (exploring how various etiologies affect different organs)	4.44	0.62	0.14	94.44%			
1.11 Definition of TCM health preservation (traditional theories and methods for body-mind cultivation and disease prevention)	4.56	0.70	0.15	88.89%			
1.12 Theoretical basis of TCM health preservation (principles of yin-yang, five elements, and the "unity of nature and humanity")	4.61	0.50	0.11	100.00%			

1.13 Five dimensions of TCM health preservation (sleep and wake routines, diet, emotion, mind-body exercise, and meridian)	4.67	0.49	0.10	100.00%			
1.14 Safety, target population, and precautions (emphasizing natural and gentle regulation with high safety; suitable for most insomnia patients seeking non-pharmacological methods; requires individualized adjustments and professional guidance for severe cases or special groups like pregnant women)	4.50	0.51	0.11	100.00%			
**Domain 2. Emotional regulation**	4.72	0.57	0.12	94.44%			
2.1 Definition of emotional regulation (cultivating virtue and spirit to enhance psychological well-being based on TCM theories)	4.61	0.50	0.11	100.00%			
2.2 Essentials of pre-sleep emotional regulation	4.61	0.50	0.11	100.00%		Merged with 2.2.1, 2.2.2 and 2.2.3 in round 4	
2.2.1 Avoiding extreme emotions before bed (preventing "heart-spirit" disturbance caused by intense emotional fluctuations)	4.72	0.46	0.10	100.00%			
2.2.2 Transforming the mindset and overcoming one emotion with another	4.39	0.70	0.16	88.89%			
2.2.3 Avoiding the use of electronic products before bed (laptops or smartphones)	4.78	0.43	0.09	100.00%			
2.3 Introduce methods of emotional regulation	4.56	0.51	0.11	100.00%		Merged with 2.3.2 and 2.3.3 in round 4	
2.3.1 Distraction method (shifting focus through hobbies (e.g., arts, sports) to resolve negative emotions)	3.78	0.94	0.25	66.67%	Failed	Deleted	
2.3.2 Self-regulation (regulating and managing emotions, preventing excessive emotional reactions, and maintaining psychological balance as beneficial for improving sleep)	4.44	0.62	0.14	94.44%			
2.3.3 Catharsis method (releasing suppressed emotions through communication or healthy venting)	4.33	0.69	0.16	88.89%			
2.3.4 *Qi* regulation method (harmonizing *Zang-fu* functions and "spirit" via traditional exercises, e.g., body-breath-mind adjustment)	4.50	0.62	0.14	94.44%			
**Domain 3. Dietary regulation**	4.72	0.46	0.10	100.00%			
3.1 TCM definition of dietary regulation (selecting and harmonizing foods based on TCM theories to balance yin-yang and prevent disease)	4.61	0.61	0.13	94.44%			
3.2 TCM basic theories of dietary regulation (including the "four natures" of food: cold, cool, warm, and hot; and the "five flavors": sour, bitter, sweet, pungent, and salty)	4.83	0.38	0.08	100.00%			
3.3 Functions of dietary regulation (nourishing the body, delaying aging, and strengthening	4.56	0.51	0.11	100.00%			

resistance against pathogenic factors)							
3.4 Principles of dietary regulation	4.39	0.70	0.16	88.89%		Merged (with 3.4.1, 3.4.2, 3.4.3)	
3.4.1 Balanced diet with appropriate combination of foods (emphasis on diverse food groups and rational nutritional structure (e.g., food guide pagoda))	4.06	0.80	0.20	72.22%	Failed	Merged (with 3.4)	
3.4.2 Individualized dietary strategies (tailoring diets according to personal constitution, seasonal changes, and geographical environment)	4.50	0.62	0.14	94.44%		Merged (with 3.4)	
3.4.3 Moderation and dietary taboos (regulating meal timing and quantity, while avoiding irritants like caffeine or heavy food before bed)	4.72	0.46	0.10	100.00%		Merged (with 3.4)	
3.5 Selection of dietary ingredients [proper use of five grains (staples), vegetables, fruits, and meats; appropriate use of seasonings]	4.56	0.62	0.14	94.44%			
3.6 Syndrome differentiation-based diet (medicinal food based on insomnia types)	4.61	0.50	0.11	100.00%			
3.7 Health-preserving herbal teas (using herbal tea formulas, such as wolfberry and chrysanthemum, to clear fire and nourish yin)	4.39	0.61	0.14	94.44%			
3.8 Precautions for dietary regulation	4.44	0.78	0.18	83.33%			
**Domain 4. Sleep-wake routines**	4.72	0.46	0.10	100.00%			
4.1 Definition of sleep-wake routines (regulating daily routines to align with natural and physiological rhythms)	4.67	0.49	0.10	100.00%			
4.2 Theory of daily living (harmonizing with nature, balancing work and rest, and optimizing the living environment)	4.72	0.46	0.10	100.00%			
4.3 The "Midday-Midnight Circadian Qi Flow" (*Zi-Wu-Liu-Zhu*) [sleeping during *Zi* (23:00–01:00) and napping during *Wu* (11:00–13:00, < 1 h)], even if a perfect alignment with nature isn't possible, keeping a regular sleep routine is essential and should be maintained as much as possible)	4.56	0.70	0.15	88.89%			
4.4 TCM herbal aromatherapy for insomnia tailored to various body types (with hands-on sachet making)	4.28	0.57	0.13	94.44%			
4.5 Taking a warm bath or soaking feet before bedtime	4.33	0.59	0.14	94.44%			
4.6 Sleep environment and selection of bedding (ensuring a quiet environment with comfortable pillows, mattresses, and sleepwear)	4.22	0.55	0.13	94.44%			
4.7 Methods for judging sleep quality (assessing sleep onset speed, frequency of waking, depth of sleep, and morning alertness)	4.17	0.62	0.15	88.89%			

4.8 Formulation of personal sleep plans (developing a scientific daily sleep schedule tailored to individual needs)	4.50	0.71	0.16	88.89%			
**Domain 5. Mind–body exercise**	4.50	0.62	0.14	94.44%			
5.1 Definition of mind–body exercise (integrating body movements and breathing to achieve “unity of body and spirit”)	4.56	0.51	0.11	100.00%			
5.2 Characteristics and functions (harmonizing qi and blood and improving Zang-fu functions through dynamic/static balance)	4.56	0.51	0.11	100.00%			
5.3 Key essentials (exercise and regulation of body, *qi*, spirit)	4.28	0.75	0.18	83.33%			
5.4 Principles of practice (moderation, individualization, and persistent, step-by-step progress)	4.50	0.62	0.14	94.44%			
5.5 Common exercise practices (e.g., *Tai Chi* and *Baduanjin*) introduced via video	4.28	0.75	0.18	83.33%			
5.6 Leading participants to practice *Baduanjin* in class	4.11	0.68	0.16	83.33%			
5.7 Pre-sleep relaxation exercises (gentle movements like yoga and meditation to calm the mind)	4.06	1.11	0.27	72.22%	Failed	Revised (Self-directed meditation may counterproductively worsen insomnia)	
5.8 Precautions and individualized suggestions (adjusting intensity based on constitution and avoiding vigorous exercise before bed)	4.56	0.51	0.11	100.00%			
**Domain 6. Acupoint massage**	4.50	0.62	0.14	94.44%			
6.1 Practical in-class training of key acupoints [hands-on practice for easily operable points: *Baihui* (GV20), *Shenmen* (HT7), *Neiguan* (PC6), and *Yintang* (GV29), operational precautions and safety guidelines (managing pressure/timing; special advice for pregnant women or those with skin conditions]	4.67	0.49	0.10	100.00%			1 item: Definition and functions of acupoint massage
6.2 Bedtime foot shaking: energy balance and mind calming	3.83	0.86	0.22	66.67%	Failed	Deleted	
6.3 Hand-on-navel and *qi*-sinking-to-*dantian*	4.28	0.57	0.13	94.44%			
6.4 *Yongquan* massage: *yin* restoration and sleep quality	4.22	0.73	0.17	83.33%			

a SD: Standard Deviation;

b CV: Coefficient of Variation;

c CLA: Consensus Level of Agreement;

Consensus Status: “Failed” indicates the item did not simultaneously meet the pre-defined thresholds (M $\geqslant$ 4.0, CV < 0.25, and CLA $\geqslant$ 80%);

Action Taken: Includes items that were revised, merged, or deleted during the Delphi rounds.

Table S4. Results of Delphi round 4: Importance ranking of items within each domain

Content	Sum of Ranks	Mean Rank	SD	Important ranking
**Domain 1. Introduction to TCM-L framework**				
TCM definition of insomnia (dissatisfaction with sleep quantity/quality without physical disease)	71	3.94	3.24	1
Etiology of insomnia (key factors including dietary irregularities, emotional distress, and imbalance between work and rest)	93	5.17	2.15	2
TCM sleep theories (exploring how sleep is regulated by the circadian balance of yin-yang, internal energy circulation, and the governance of the mind)	98	5.44	3.29	3
Theoretical basis of TCM health preservation (principles of yin-yang, five elements, and the “unity of nature and humanity”)	105	5.83	4.26	4
Definition of TCM health preservation (traditional theories and methods for body-mind cultivation and disease prevention)	109	6.06	5.00	5
Five-dimensional content of TCM health preservation (sleep and wake routines, diet, emotion, mind–body exercise, and meridian)	118	6.56	4.09	6
Pathogenesis of insomnia (key mechanism involving heart–spirit instability, related to the heart, liver, spleen, and kidney)	125	6.94	2.21	7
Relationship between the *Zang-fu* organs and insomnia (exploring how various etiologies affect different organs)	139	7.72	2.37	8
Self-diagnosis of TCM body constitution types of insomnia (common constitutions including qi deficiency, yin deficiency, etc.; individuals with different constitutions will exhibit different clinical manifestations when experiencing insomnia)	152	8.44	2.87	9
TCM non-pharmacological therapies and multidimensional interventions (e.g., acupoint stimulation; external herbal treatments; moxibustion; massage; emotional regulation; exercise; and multidimensional interventions will integrating physiological, psychological, and lifestyle adjustments)	166	9.22	4.39	10
TCM treatment principles (with regulation of *Zang-fu*, harmonization of qi and blood, and calming of the mind)	166	9.22	3.19	10
Safety, target population, and precautions (emphasizing natural and gentle regulation with high safety; suitable for most patients with insomnia seeking non-pharmacological methods; requires individualized adjustments and professional guidance for severe cases or special groups like pregnant women)	174	9.67	4.70	12
Group discussion on prevention methods (maintaining emotional stability, regular lifestyle, and healthy sleep hygiene)	183	10.17	3.11	13
TCM therapies for specific pathogenesis (applying targeted techniques based on syndrome types; e.g., using acupoint massage and dietary therapy to nourish the heart and spleen for patients with heart–spleen deficiency)	191	10.61	3.18	14
Kendall’s W = 0.249 $\chi^2 = 58.36$, *P < 0.001.*				
**Domain 2. Emotional regulation**				
Definition of emotional regulation (cultivating virtue and spirit to enhance psychological well-being based on TCM theories)	35	1.94	1.76	1
Self-regulation (regulating and managing emotions, preventing excessive emotional reactions, and maintaining psychological balance as beneficial for improving sleep)	39	2.17	1.34	2
Catharsis method (releasing suppressed emotions through communication or healthy venting)	63	3.50	1.42	3
Qi regulation method (harmonizing *Zang-fu* functions and “spirit” via traditional exercises, e.g., body-breath-mind adjustment)	71	3.94	1.55	4

Avoiding the use of electronic products before bed (laptops or smartphones)	87	4.83	2.48	5
Avoiding extreme emotions before bed (preventing “heart–spirit” disturbance caused by intense emotional fluctuations)	89	4.94	2.39	6
Transforming the mindset and overcoming one emotion with another	89	4.94	2.18	6
Kendall’s W = 0.503, $\chi^2 = 54.33$*, P < 0.001.*				
**Domain 3. Dietary regulation**				
TCM basic theories of dietary regulation (including the “four natures” of food: cold, cool, warm, and hot; and the “five flavors”: sour, bitter, sweet, pungent, and salty)	42	2.33	1.91	1
Principles of dietary regulation (individualized dietary strategies, moderation, and dietary taboos)	56	3.11	1.45	2
Functions of dietary regulation (nourishing the body, delaying aging, and strengthening resistance against pathogenic factors)	59	3.28	2.02	3
TCM definition of dietary regulation (selecting and harmonizing foods based on TCM theories to balance yin-yang and prevent disease)	60	3.33	2.81	4
Dietary strategies for different constitutions	63	3.50	1.76	5
Selection of dietary ingredients [proper use of five grains (staples), vegetables, fruits, and meats; appropriate use of seasonings]	88	4.89	1.88	6
Health-preserving herbal teas (using herbal tea formulas, such as wolfberry and chrysanthemum, to clear fire and nourish yin)	100	5.56	1.85	7
Precautions for dietary health preservation	101	5.61	2.15	8
Kendall’s W = 0.392, $\chi^2 = 49.33$*, P < 0.001.*				
**Domain 4. Sleep-wake routines**				
Theory of sleep-wake routines (harmonizing with nature, balancing work and rest, and optimizing the living environment)	44	2.44	1.95	1
Definition of daily living health preservation (regulating daily routines to align with natural and physiological rhythms)	48	2.67	2.68	2
Methods for judging sleep quality (assessing sleep onset speed, frequency of waking, depth of sleep, and morning alertness)	64	3.56	1.89	3
Formulation of personal sleep plans (developing a scientific daily sleep schedule tailored to individual needs)	78	4.33	2.30	4
The “Midday-Midnight Circadian *Qi* Flow” (*Zi-Wu-Liu-Zhu*) [sleeping during *Zi* (23:00–01:00) and napping during *Wu* (11:00–13:00, < 1 h)]	80	4.44	1.89	5
TCM herbal aromatherapy for insomnia tailored to various body types (with hands-on sachet making)	94	5.22	1.63	6
Taking a warm bath or soaking feet before bedtime	102	5.67	1.37	7
Sleep environment and selection of bedding (ensuring a quiet environment with comfortable pillows, mattresses, and sleepwear)	102	5.67	1.37	7
Kendall’s W = 0.300 $\chi^2 = 35.75$*, P < 0.001.*				
**Domain 5. Mind–body exercise**				
Characteristics and functions (harmonizing *qi* and blood and improving *Zang-fu* functions through dynamic/static balance)	56	3.11	1.28	1
Definition of mind–body exercise (integrating body movements and breathing to achieve “unity of body and spirit”)	59	3.28	2.56	2
Key essentials (exercise and regulation of body, *qi*, and spirit)	59	3.28	1.49	2
Principles of practice (moderation, individualization, and persistent, step-by-step progress)	62	3.44	1.25	4
Leading participants to practice *Baduanjin* in class	65	3.61	2.45	5
Common exercise practices (e.g., *Tai Chi* and *Baduanjin*) introduced via video	83	4.61	2.25	6

Pre-sleep relaxation exercises (gentle movements like yoga to calm the mind)	83	4.61	2.33	6
Precautions and individualized suggestions (adjusting intensity based on constitution and avoiding vigorous exercise before bed)	87	4.83	2.20	8
Kendall's W = 0.273, $\chi^2 = 34.389$*, P < 0.001.*				
**Domain 6. Acupoint massage**				
Practical in-class training of key acupoints [hands-on practice for easily operable points: *Baihui* (GV20), *Shenmen* (HT7), *Neiguan* (PC6), and *Yintang* (GV29), operational precautions and safety guidelines (managing pressure/timing; special advice for pregnant women or those with skin conditions]	24	1.33	0.69	1
Definition and functions of acupoint massage	24	1.33	0.69	1
Hand-on-navel and *Qi*-sinking-to-*Dantian*	41	2.28	1.02	3
Yongquan massage: *yin* restoration and sleep quality	42	2.33	0.84	4
Kendall's W = 0.272, $\chi^2 = 14.667$*, P = 0.002*				

Table S5. Results of Delphi round 4: Recommended curriculum sequence of the six domains

Module name	Sum of ranks	Mean rank	SD	Rank
**Domain 1:** Introduction to TCM-L framework	26	1.44	0.98	1
**Domain 4:** Sleep-wake routines	55	3.06	1.35	2
**Domain 3:** Dietary regulation	62	3.44	1.29	3
**Domain 2:** Emotional regulation	63	3.50	1.46	4
**Domain 6:** Acupoint massage	86	4.78	1.52	5 (Tie)
**Domain 5:** Mind – body exercise	86	4.78	1.17	5 (Tie)

Expert consensus: *Kendall's W* = 0.717, $\chi^2 = 64.54$, $P < 0.001$
